# Contrasting patterns of leptomeningeal and parenchymal gadolinium extravasation in cerebral amyloid angiopathy: An MRI-based evaluation

**DOI:** 10.1177/17474930261424089

**Published:** 2026-02-05

**Authors:** S Voigt, JJA de Jong, PHM Voorter, EA Koemans, MR Schipper, MC van der Plas, K Kaushik, RGJ van der Zwet, R van Dort, H van den Brink, SJ van Veluw, MJP van Osch, MAA van Walderveen, WH Backes, MJH Wermer, WM Freeze

**Affiliations:** 1 Department of Neurology, Leiden University Medical Center, Leiden, The Netherlands; 2 Department of Radiology, Leiden University Medical Center, Leiden, The Netherlands; 3 Department of Radiology & Nuclear Medicine, Maastricht University Medical Center, Maastricht, The Netherlands; 4 Mental Health and Neuroscience Research Institute (MHeNs), Maastricht University, Maastricht, The Netherlands; 5 Department of Neurology, J. Philip Kistler Stroke Research Center, MGH, Boston, MA, USA; 6 MassGeneral Institute for Neurodegenerative Disease, MGH, Boston, MA, USA; 7 Cardiovascular Research Institute Maastricht (CARIM), Maastricht University, Maastricht, The Netherlands; 8 Department of Neurology, University Medical Center Groningen, Groningen, The Netherlands

**Keywords:** Intracerebral hemorrahage, MRI, neurology, hemorrhage, brain microbleeds, cerebral hemorrhage

## Abstract

**Background:**

Previous studies suggest that blood–brain barrier (BBB) disruption may play a role in the pathophysiology of vessel rupture in cerebral amyloid angiopathy (CAA). Here, in a cross-sectional cohort study, we apply contrast-enhanced 3 Tesla magnetic resonance imaging (MRI) to test the hypothesis that the BBB is damaged in patients with CAA and to determine whether BBB leakage is associated with hemorrhagic brain injury in CAA.

**Methods:**

Parenchymal BBB leakage rate (K_i_) was assessed in the cortex and white matter with dynamic contrast-enhanced (DCE)-MRI and quantified with pharmacokinetic modeling. Leptomeningeal BBB leakage was assessed visually on post-contrast heavily T2-weighted fluid attenuation inversion recovery (FLAIR) images. Cortical cerebral microbleeds (CMBs) and cortical superficial siderosis (cSS) were assessed on susceptibility-weighted images. Analyses included descriptive statistics, group comparisons using the Mann–Whitney U test and Spearman’s rank correlation for associations with imaging markers.

**Results:**

In total, 25 patients with a clinical diagnosis of probable CAA without prior intracerebral hemorrhage and 19 age- and sex-matched controls were included. In patients with CAA, BBB leakage rates were lower in the cortex (4.2 × 10^−4^ min^−1^ vs 5.6 × 10^−4^ min^−1^; p = 0.004) and in the white matter (1.2 × 10^−4^ min^−1^ vs 2.1 × 10^−4^ min^−1^; p < 0.001) compared with controls. The presence of leptomeningeal enhancement was higher in patients with CAA (68%) compared with controls (47%) (p = 0.007). Within the group with CAA, we did not find a correlation between the number of cortical leakage and cortical CMBs (Spearman’s ρ = 0.06, p = 0.79) or the number of foci of leptomeningeal enhancement and cSS hemisphere score (Spearman’s ρ = 0.30, p = 0.15).

**Discussion:**

Our results suggest that global parenchymal gadolinium extravasation across the BBB is lower in patients with CAA compared with controls. This observation can be explained in terms of limited capillary blood perfusion and/or raising the possibility that vascular amyloid-β deposition impairing molecular transport across the BBB. In contrast, focal leptomeningeal enhancement was higher in CAA, reflecting vessel wall infiltration.

## Introduction

Cerebral amyloid angiopathy (CAA) is a major cause of intracerebral hemorrhage (ICH) and cognitive decline in the elderly.^1^ It is characterized by deposition of amyloid-β in the leptomeningeal arteries, cortical arterioles, and capillaries.^1^ CAA is affecting the cerebral vessels, resulting in several hemorrhagic manifestations, such as ICH, cerebral microbleeds (CMBs), and cortical superficial siderosis (cSS).^2^

The mechanisms behind hemorrhage development are not yet fully understood. Previous neuropathological and experimental rodent studies suggest that vessel rupture may result from a sequence of events, including increased accumulation of amyloid-β, vascular dysfunction, impaired perivascular clearance, and eventual breakdown of the vessel wall.^3–7^ This has led to increased interest in cerebrovascular reactivity, cerebral blood flow and pulsatility, as well as blood–brain barrier (BBB) integrity.^8^

The BBB is a very selectively permeable interface between the bloodstream and the brain parenchyma, formed by endothelial cells connected via tight junctions, supported by pericytes and astrocytic endfeet.^9^ It plays a crucial role in maintaining cerebral homeostasis by regulating the exchange of molecules. In CAA, several studies suggest that amyloid-β deposition in vessel walls may compromise BBB integrity by inducing endothelial dysfunction, inflammatory responses, and degeneration of vascular components.^10–12^ This could lead to increased permeability, potentially contributing to disease progression and hemorrhagic manifestations. Dynamic contrast-enhanced MRI (DCE-MRI) allows for in vivo assessment of BBB integrity by visualizing and quantifying the extravasation of gadolinium-based contrast agents into the brain parenchyma and leptomeninges, making it a valuable tool to investigate subtle BBB dysfunction in CAA.

In this study, we applied contrast-enhanced MRI to assess gadolinium leakage in the parenchyma as well as the leptomeninges. We hypothesized that BBB leakage would be increased in the parenchyma and leptomeninges of patients with CAA compared with controls, and that BBB leakage would be associated with hemorrhagic brain injury. As no treatment is currently available to prevent hemorrhage in CAA, there is a strong need for early markers of disease progression. Insights into BBB dysfunction may improve our understanding of hemorrhage formation and help identify patients at risk for future bleeding, potentially enabling early intervention.

## Methods

### Study participants

We included participants diagnosed with probable CAA according to the Boston criteria version 2.0,^2^ from the FOCAS study: our longitudinal natural history study on CAA disease progression. Participants were prospectively recruited from our specialized CAA clinic according to predefined inclusion and exclusion criteria that were applied consistently. Participants with a history of ICH were excluded to avoid ICH-related secondary BBB disruption in the study.^13,14^ Other exclusion criteria were elevated risk associated with the administration of contrast agents if they had severely impaired renal function—defined as severe renal insufficiency (estimated glomerular filtration rate (eGFR) < 30 mL/min/1.73 m^2^) or conditions such as nephrogenic systemic fibrosis or nephrogenic fibrosing nephropathy. In case of uncertainty about the safety of contrast administration or MRI suitability, inclusion was discussed with an experienced neuroradiologist (M.A.A.v.W.) to ensure participant safety. We included age- and sex-matched control participants without any known neurologic disease, to compare BBB leakage of patients with CAA to a reference group. The control participants were recruited via an advertisement in a local newspaper. This study was approved by our local medical ethics committee (The Medical Ethics Review Committee Leiden—The Hague—Delft, Leiden University Medical Center, number P17.259), and all participants gave written informed consent prior to participation.

### Study procedures

During the study visit, we collected baseline characteristics on vascular risk factors (hypertension, hypercholesterolemia, body weight, and smoking) and administered two neuropsychological screening tests: the Mini-Mental State Examination (MMSE) and Montreal Cognitive Assessment (MoCA). A venous blood sample was collected to assess renal function (eGFR) and blood creatinine levels prior to the MRI scan. Individual hematocrit blood levels were measured to optimize the calculation of gadolinium levels within the blood plasma in the DCE-MRI analysis. Furthermore, a dynamic gadolinium contrast-enhanced MRI was performed (Supplementary Methods; MRI acquisition and brain segmentation).

### BBB leakage

As described previously,^15–18^ leakage from the parenchymal vessels was assessed by applying pharmacokinetic modeling using the fast and slow DCE sequence images. For both the fast and slow sequences, a reference image was calculated using the average of the pre-contrast images in each sequence, which was used for motion correction. In addition, the fast sequence images were spatially co-registered to the slow sequence images using a single, linear spatial transformation. The reference image of the slow sequence was registered to the T1-weighted images, and the inverse of the obtained transformation matrix was used to transform the T1-weighted images, including the masks for the tissue regions, to slow sequence space.

To obtain a vascular input function (VIF) for each participant, at least 20 adjacent voxels in the superior sagittal sinus were manually selected (J.d.J.).^19,16^ Conversion of the MR signal to gadolinium concentration was applied for the VIF and the brain tissue by assuming a linear relationship between T1 relaxation rate changes and gadolinium concentration and using the tissue relaxation times obtained from the pre-contrast T1 map.^15,17^ The concentration in the blood plasma was calculated from the VIF and corrected for individual hematocrit.

The graphical Patlak approach^20^ was applied to using the gadolinium concentration-time curves in brain tissue and blood plasma for voxel-wise assessment of parenchymal BBB leakage, characterized by K_i_ (min^−1^), the leakage rate from the blood plasma to the parenchyma as a measure of contrast agent leakage, and V_p_ (−), the volume fraction of blood plasma serving as an indicator of blood perfusion. Per tissue region (gray matter (GM), normal-appearing white matter (NAWM), and white matter hyperintensities (WMHs)), the leakage and blood perfusion were summarized by the mean K_i_ and median V_p_, respectively.

Leakage at the level of the leptomeningeal vasculature was determined based on visual assessment of the pre- and post-contrast heavily T2-weighted fluid attenuation inversion recovery (FLAIR) images by two experienced raters (E.A.K. and W.M.F.) according to previously published criteria.^21–23^ In short, the number and location (lobe) of leptomeningeal enhancement were assessed. Leptomeningeal enhancement was classified as focal (distinct spots of enhancement that appear as nodular or linear shape) or sulcal (more diffuse enhancement typically occurring within the sulcal CSF, and filling at least 50% of the sulcal volume) when present on post-contrast images but not on pre-contrast images. Foci of enhancement adjacent to the falx cerebri (dural enhancement) were not included,^24^ as well as foci with a halo-like appearance or surrounding the superior sagittal sinus (paravenous enhancement).^25^ Scans with low image quality were excluded, defined by the presence of substantial motion artifacts or a poor contrast-to-noise ratio that impeded reliable evaluation of leptomeningeal enhancement.

### Neuroradiological markers

Two experienced raters (S.V. and M.C.v.d.P.) assessed the following CAA-related MRI markers according to the STRIVE criteria 2.0: cSS, cortical CMBs, deep microbleeds, and perivascular spaces in the centrum semiovale (CSO-PVS) to evaluate the burden of CAA-related pathology.^26^

### Sample size calculation

Based on prior post-mortem findings (r ⩾ 0.77),^3^ we expected a correlation of at least r ⩾ 0.6 between cortical leakage and the number of CMBs. To detect this correlation (α = 0.05, power = 80%), a minimum of 19 participants with CAA was required. In addition, to detect a difference in leakage prevalence between patients with CAA (expected ⩾ 65%) and controls (expected 19%) on post-contrast FLAIR,^21^ 17 participants per group were needed. To account for potential data loss (e.g. motion artifacts), we aimed to include 25 participants with sporadic CAA and 20 age- and sex-matched controls.

### Statistical analyses

Descriptive statistics were used for baseline characteristics. Based on the type of data, appropriate statistical tests for comparisons between the groups were used: we used the Mann–Whitney U test for non-parametric K_i_ values, the volume fraction of blood plasma (V_p_), and leptomeningeal enhancement. The correlation between cortical BBB leakage rate and cortical CMBs, leptomeningeal enhancement, and cSS was assessed using Spearman's rank correlation analysis. Outliers were included in the analysis but excluded from the scatter plots to improve readability. Interrater reliability was assessed using a two-way intraclass correlation coefficient (ICC) model for agreement between two raters. All statistical analyses were performed with SPSS v29.^27^

## Results

We initially included 25 participants with probable CAA (mean age 70.8 years, 60% women) and 20 control participants (mean age 70.6 years, 47% women). One control participant was subsequently excluded due to an incidental finding of a meningioma, resulting in a final control group of 19 participants. The prevalence of vascular risk factors and cognition scores (measured with MMSE and MoCA) was similar between the two groups (Table 1). Fifteen (60%) patients with CAA had cSS versus 0 (0%) of the control participants. All patients with CAA had lobar CMBs, with a median of 60 (min. 5 − max. 1433). Five control participants had lobar CMBs, with a median of 3 (min. 1 − max. 7). The median WMH volume was 14 cm^3^ in patients with CAA versus 4.4 cm^3^ in the control participants (Table 1).

**Table 1.** Characteristics of the participants.

	Patients with CAA (n = 25)	Controls (n = 19)
Age, mean (SD)	70.8 (7.2)	70.6 (6.8)
Women, n (%)	15 (60%)	9 (47%)
Mixed type^a^, n (%)	8 (32.0%)	0 (0%)
Mean distribution CMB, lobar:deep	100:0.3	0 (0%)
Hypertension^b^, n (%)	10 (40%)	6 (32%)
Hypercholesterolemia^c^, n (%)	8 (32%)	6 (32%)
Overweight, n (%)	5 (20%)	4 (21%)
Smoking (current or stopped), n (%)	15 (60%)	11 (58%)
MMSE score, median (IQR)	28 (26-30)	29 (28-30)
MoCA score, median (IQR)	27 (24-30)	27 (25-28)
Hematocrit, median (IQR)	0.41 (0.40-0.45)	0.40 (0.40-0.43)
eGFR, median (IQR)	68 (63-72)	67 (58-74)
APOE genotype		
ε2ε2, n (%)	2 (8%)	0 (0%)
ε2ε3, n (%)	0 (0%)	2 (10%)
ε2ε4, n (%)	1 (4%)	0 (0%)
ε3ε3, n (%)	7 (28%)	14 (74%)
ε3ε4, n (%)	5 (20%)	3 (16%)
ε4ε4, n (%)	9 (36%)	0 (0%)
Intracranial volume (ICV) in cm^3^, median (IQR)	1449 (1405-1635)	1440 (1396-1602)
Total brain volume as percentage of ICV, mean (min-max)	69 (61-74)	71 (65-76)
Cortical superficial siderosis (cSS), n (%)	15 (60%)	0 (0%)
Focal cSS, n (%)	3 (12%)	0 (0%)
Disseminated cSS, n (%)	12 (48%)	0 (0%)
Hemisphere score, n (%)		
1	3 (12%)	0 (0%)
2	3 (12%)	0 (0%)
3	2 (8%)	0 (0%)
4	7 (28%)	0 (0%)
Lobar microbleeds, n (%)	25 (100%)	5 (26%)
Number, median (min-max)	60 (5-1433)	3 (1-7)
WMH volume in cm^3^, median (IQR)	14 (4.5-27.5)	4.4 (1.7-9.8)
CSO-PVS score		
1	0 (0%)	0 (0%)
2	3 (12%)	1 (5%)
3	7 (28%)	9 (47%)
4	14 (56%)	9 (47%)

^a^CAA diagnosis was changed to mixed-type small vessel disease due to deep bleeds previously unobserved on lower field MRI. ^b^Hypertension was defined as ⩾140/90 mm Hg and/or use of antihypertensive drugs. ^c^Reported in medical history and/or use of statin.

In patients with CAA, we found lower K_i_ values in the cortex (4.2 × 10^−4^ min^−1^ vs 5.6 × 10-4 min^−1^; p = 0.004), white matter (1.2 × 10^−4^ min^−1^ vs 2.1 × 10^−4^ min ^−1^; p < 0.001), NAWM (1.2 × 10^−4^ min^−1^ vs 2.1 × 10^−4^ min ^−1^; p < 0.001), and WMH (2.4 × 10^−4^ min^−1^ vs 3.4 × 10^−4^ min^−1^; p = 0.002) compared with control participants (Figure 1 and Table 2). Also lower V_p_ values were obtained in the cortex (0.007 vs 0.011; p = 0.038), white matter (0.002 vs 0.004; p = 0.001), NAWM (0.002 vs 0.004; p = 0.001), and WMH (0.004 vs 0.008; p = 0.001) in patients with CAA compared with controls (Table 2). There was no correlation between cortical leakage and the number of cortical CMBs (Spearman’s ρ = 0.06, p = 0.79) in patients with CAA. These results remained similar after excluding controls with CMBs from the analysis. A sensitivity analysis excluding patients with mixed-type small vessel disease (n = 8) yielded similar results, indicating that our main findings were robust to this subgroup.

**Figure 1.**
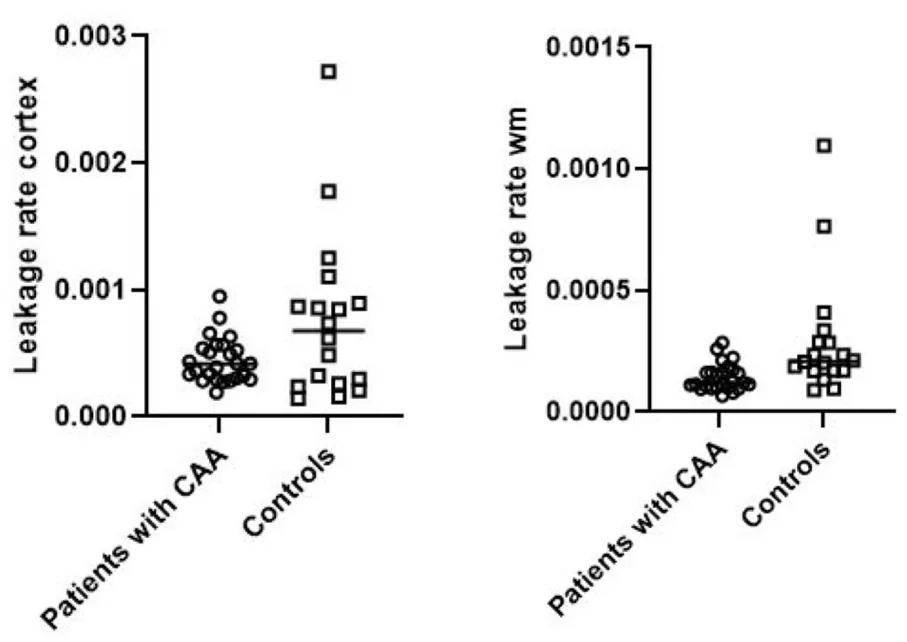
Boxplots of BBB leakage rate in cortex and white matter (wm). In both boxplots, one outlier has been excluded from each to maintain readability: these outliers represent two different participants (cortex; control participant with K_i_ of 0.0092, wm; control participant with K_i_ of 0.0026). These data points were, however, included in all statistical analyses.

**Table 2.** BBB leakage rate, Vp and leptomeningeal enhancement in patients with CAA and control participants.

	Patients with CAA (n = 25)	Controls (n = 19)	p-value
BBB leakage rate (K_i_) 10^−4^ per min^−1^			
Cortex, median (IQR)	4.2 (3.0-5.5)	5.6 (4.6-8.5)	0.004
White matter, median (IQR)	1.2 (1.0-1.7)	2.1 (1.7-3.4)	< 0.001
Normal-appearing white matter, median (IQR)	1.2 (1.0-1.7)	2.1 (1.7-3.4)	< 0.001
White matter hyperintensities, median (IQR)	2.4 (1.4-3.3)	3.4 (2.6-5.7)	0.002
Vp 10^−2^ (the volume fraction of blood plasma as a measure of blood perfusion)			
Cortex, median (IQR)	0.70 (0.60-1.00)	1.10 (0.60-1.20)	0.038
White matter, median (IQR)	0.20 (0.10-0.30)	0.40 (0.30-0.60)	0.001
Normal-appearing white matter, median (IQR)	0.20 (0.10-0.30)	0.40 (0.30-0.60)	0.001
White matter hyperintensities, median (IQR)	0.40 (0.30-0.70)	0.80 (0.40-1.10)	0.005
Leptomeningeal enhancement			
Leptomeningeal enhancement present, n (%)	17 (68%)	9 (47%)	0.078
Total leptomeningeal enhancement foci, median (min-max)	2 (1-14)	1 (1-2)	0.007
Focal present, n (%)	16 (94%)	8 (89%)	0.073
Focal count, median (Q1-Q3)	2 (1-7)	1 (1-1)	0.011
Sulcal present, n (%)	7 (41%)	2 (22%)	0.118
Sulcal count, median (Q1-Q3)	1 (1-2)	1 (1-1)	0.108

For the assessment of leptomeningeal enhancement, two patients with CAA were excluded due to low scan quality. Seventeen (68%) patients with CAA had focal and/or sulcal leptomeningeal enhancement versus nine (47%) control participants (p = 0.078). The median number of total foci was higher in patients with CAA (p = 0.007) (Table 2). Patients with CAA had a higher median focal count (p = 0.011) (Table 2 and Figure 2). Seven (41%) patients with CAA with leptomeningeal enhancement had sulcal presence versus two (22%) of the control participants (p = 0.118) (Table 2). Notably, the control participants with sulcal leptomeningeal enhancement both had cortical CMBs (3 and 7 CMBs). When we excluded the control participants with CMBs, no sulcal leptomeningeal enhancement was observed in the remaining control participants (41% vs 0%, p = 0.01). Within patients with CAA, we did not find a correlation between the number of foci of leptomeningeal enhancement and cSS hemisphere score (Spearman’s ρ = 0.30, p = 0.15) nor for the presence of sulcal enhancement and cSS hemisphere score (p = 0.062). The ICC for interrater agreement of the number of foci of leptomeningeal enhancement between the two raters was 0.81 (95% confidence interval (CI) = 0.666–0.896), indicating substantial agreement.

**Figure 2.**
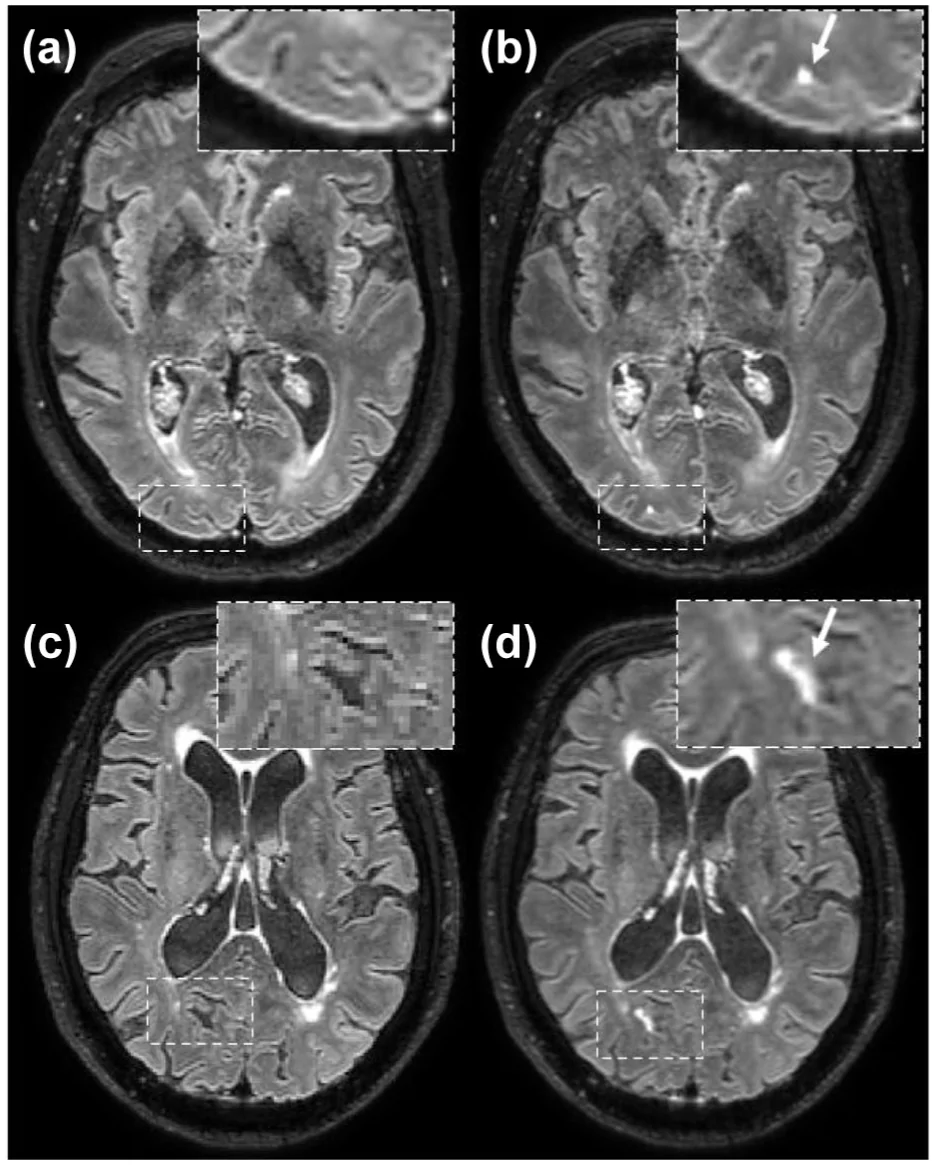
Focal and sulcal leptomeningeal blood–brain barrier leakage. Precontrast heavily 3D T2-weighted FLAIR images show normal-appearing cerebrospinal fluid in the subarachnoid space (a and c). The corresponding postcontrast FLAIR images acquired approximately 16 min after contrast administration show focal (b) and sulcal (d) signal enhancement in the CSF space.

## Discussion

In this study, we found lower leakage rates and blood plasma volume fractions in the cortex, NAWM, and WMH of patients with CAA versus control participants. BBB leakage rate in the cortex was not associated with cortical CMB burden or presence of cSS in patients with CAA. The total number of foci of CSF enhancement due to leakage from the leptomeninges was higher in patients with CAA.

Our results suggest that gadolinium extravasation from the microcirculation to the parenchyma as well as tissue perfusion is reduced in patients with CAA compared with controls. The lower leakage rate in patients with CAA does not correspond with previous findings in other cerebral small vessel disease (cSVD) types.^28,29^ Previous studies in patients with age-related cSVD show higher as well as similar leakage rates in comparison to controls. And mostly higher,^28,30^ but also lower leakage rates have been reported in WMH versus NAWM in patients with age-related cSVD.^29,31^ Our finding of lower leakage in CAA-type cSVD compared with controls raises the possibility that vascular amyloid-β deposition interferes with normal BBB functioning. Upon reflection, one possible explanation might be that the deposition of amyloid and potentially other proteins such as fibrin may act as a form of “coating” on the vascular wall,^3^ depriving free exchange of molecules between the intravascular and extravascular space.

Lower perfusion has been associated with increasing total cSVD burden, reflected by reductions in both global and regional cerebral blood flow.^32^ A possible explanation might be a declined tissue perfusion due to reduced blood volume. This reduction in perfusion could result in less gadolinium delivered to the microvasculature, thereby limiting the amount available for extravasation or leakage. In addition, our findings may be partly driven by a concomitant reduction in Vp, which reflects reduced perfusion and vascular surface area. As K_i_ is influenced by both permeability and perfusion, a lower Vp could lead to an overall reduction in leakage rate even if intrinsic BBB permeability was increased. This interpretation suggests that the observed lower K_i_ may not solely indicate a “tighter” BBB, but rather a complex interplay between reduced vascular surface area, impaired perfusion, and altered permeability in CAA. The cause of such reduced perfusion remains to be clarified but may involve decreased cerebral blood flow, increased intracerebral vascular resistance (possibly from amyloid-induced vascular stiffening), or a decrease in microvascular density. For the very low permeability range, the overall leakage rate (K_i_) is determined by both vascular permeability and the available surface area for exchange. Therefore, even if permeability increases, a substantial reduction in vascular surface area—such as may occur in CAA due to vascular loss—could still lead to a net decrease in leakage rate. Supporting this notion, recent findings show decreased microvascular claudin-5 expression in CAA associated with ICH, which may reflect both endothelial barrier disruption and a reduction in functional vessel density.^33^ This highlights that lower values in CAA could reflect not only impaired delivery of contrast agent but also structural vascular degeneration limiting the capacity for both delivery and exchange.

We found a higher count of focal leptomeningeal enhancement in patients with CAA versus controls. A possible explanation for the difference in findings between the gadolinium leakage in parenchyma and leptomeninges could be that two effects are occurring: a global effect leading to reduced leakage and a more focal effect resulting in leakage from the leptomeningeal vessels. Previous work shows more foci of contrast agent extravasation in the cortical CSF of patients with Alzheimer’s disease and mild cognitive impairment (MCI) than in control participants.^21^ Focal leptomeningeal enhancement can also be observed in healthy controls,^23^ which aligns with the findings of our study. The relatively high proportion of controls with leptomeningeal enhancement in the present study could be related to the sensitivity of the applied MRI sequence to relatively low amounts of gadolinium in the CSF, as well as the relatively high age of our control group. Although multiple studies report a positive association between focal leptomeningeal enhancement and age, the underlying physiological processes of leptomeningeal enhancement remain unknown and warrant further investigation. However, the contrast agent enhancing the MR signal of the CSF does not seem specific to CAA, as it has been observed in a wide range of other neurologic diseases as well.^23^ Sulcal enhancement was present in both patients and controls.

Interestingly, recent studies have reported sulcal FLAIR hyperintensities as an early imaging feature of CAA-related inflammation,^34^ and subarachnoid CSF hyperintensities are frequently observed in CAA on non-contrast FLAIR images acquired with 7 Tesla MRI, often colocalizing with cSS.^35^ These findings support the notion that sulcal leptomeningeal enhancement may reflect localized vascular inflammation or BBB dysfunction.

Previously, it has been suggested that BBB leakage is associated with hemorrhagic brain injury in CAA.^3,36^ For example, in patients with ICH, CSF enhancement (contrast leakage) was found and correlated with the presence of cortical CMBs.^37^ This correlation with CMBs was also found in a case report of a patient with multiple ICH, which showed development of a new microbleed at the location of a previous focus of contrast enhancement, suggesting BBB disruption as an important factor preceding the development of CMBs.^38^ Furthermore, in a post-mortem histology study, the presence of plasma proteins in the vessel wall of individual vessels was associated with the number of cortical CMBs.^3^ In the present in vivo study, however, we did not find a significant correlation between parenchymal BBB leakage rate and cortical CMBs or between leptomeningeal BBB leakage and cSS. While hemorrhagic brain pathology on MRI reflects the accumulation of brain injury over time, the assessment of parenchymal leakage with contrast-enhanced MRI represents a snapshot of the current state of BBB integrity. BBB integrity may not remain constant over time or as CAA progresses, potentially fluctuating due to factors such as inflammation or vascular remodeling. This underscores the need for longitudinal studies to further assess the correlation between BBB leakage and hemorrhagic brain injury in CAA.

A limitation of our study is that the generalizability to the broader CAA population is limited, since our study focused exclusively on participants with CAA without previous ICH. However, this exclusion was intentional to avoid confounding effects from ICH-related secondary BBB disruption.^13,14^ Future studies should therefore also include patients with CAA and previous ICH, as this represents a common and clinically relevant phenotype that may exhibit different patterns of BBB leakage. Studying this group could provide important insights into potential long-term effects of prior hemorrhage on BBB integrity and disease progression. Strengths of our study include the use of contrast-enhanced MRI to assess leakage in participants with CAA and the well-matched control participants. Also, we observed a consistent effect of reduced BBB leakage and reduced perfusion over all tissue regions. Future studies should include information on the blood perfusion and/or neurovascular coupling for a better pathophysiological understanding of the condition of the neurovascular unit. In future longitudinal studies, it would be interesting to examine the predictive value of CSF enhancement for cSS.

From a clinical perspective, these findings highlight the complexity of BBB dysfunction in CAA and suggest that reduced parenchymal leakage may reflect vessel wall remodeling due to amyloid deposition and/or reduced vascular perfusion. Distinct leptomeningeal patterns may serve as an indirect marker of CAA-related vascular pathology.

In conclusion, our study unexpectedly identified lower leakage rates and blood plasma volume fraction from the parenchymal microvessels in patients with CAA compared with controls. These observations can be explained in terms of microvascular wall deposition and rarefaction. In contrast, leakage from the larger leptomeningeal vessels was more frequently observed in patients with CAA. The broader implications and the significance of BBB leakage in the pathophysiology of CAA warrant further investigation.

## Supplemental Material

sj-docx-1-wso-10.1177_17474930261424089 – Supplemental material for Contrasting patterns of leptomeningeal and parenchymal gadolinium extravasation in cerebral amyloid angiopathy: An MRI-based evaluation Supplemental material, sj-docx-1-wso-10.1177_17474930261424089 for Contrasting patterns of leptomeningeal and parenchymal gadolinium extravasation in cerebral amyloid angiopathy: An MRI-based evaluation by S Voigt, JJA de Jong, PHM Voorter, EA Koemans, MR Schipper, MC van der Plas, K Kaushik, RGJ van der Zwet, R van Dort, H van den Brink, SJ van Veluw, MJP van Osch, MAA van Walderveen, WH Backes, MJH Wermer and WM Freeze in International Journal of Stroke
