# Supplementary material for: Contrasting patterns of leptomeningeal and parenchymal gadolinium extravasation in cerebral amyloid angiopathy: An MRI-based evaluation
Source: Int J Stroke. 2026 Feb 5;21(9):1368–76. doi: 10.1177/17474930261424089 (PMC13672920; doi:10.1177/17474930261424089)
Supplement: Supplementary file 1 [file sj-docx-1-wso-10.1177_17474930261424089.docx]

**Supplementary Methods**

*MRI acquisition*

MR images were acquired on a 3 Tesla MRI scanner (Ingenia Elition X, Philips Healthcare, Best, The Netherlands) with a 32-channel head coil. The imaging protocol consisted of a multi-echo susceptibility weighted imaging (SWI) sequence (repetition time (TR)/echo time (TE)/echospacing= 31/7.2/6.2ms, flip angle 17 degrees, 130 transversal slices and an field of view (FOV) of 230 x 190 x 130 mm with a voxel size of 0.6 x 0.6 x 1 mm), a 3D T1-weighted fast gradient echo sequence (repetition time (TR)/echo time (TE): 9.7/4.6 ms; flip angle: 8°; voxel size: 0.85 x 0.85 x 1.2 mm; FOV: 256 x 256 x 130 voxels), a 3D heavily T2-weighted fluid attenuation inversion recovery (FLAIR) sequence (TR/inversion time (TI)/TE: 4800/1650/500 ms; flip angle: 90°; isotropic voxel size: 1 mm^3^; FOV 256 x 256 x 200 voxels), and dual-time resolution DCE MRI. Dual-time DCE MRI consisted of two alternating sequences with a saturation prepulse: a fast T1 weighted saturation recovery sequence with a high temporal resolution (dynamic scan interval (DSI): 2.4 s; number of dynamic volumes: 29; TR/TE/delay time (TD): 5.3/2.5/120 ms; voxel size: 1 x 1 x 5 mm; FOV 256 x 256 x 5 voxels) to capture the steep signal changes during the initial circulations of the contrast agent (CA) in the sagittal sinus, and a slow sequence with lower temporal resolution and whole brain coverage (DSI: 30.7 s; number of dynamic volumes: 35; TR/TE/TD: 5.3/2.5/120 ms; voxel size: 1 x 1 x 2 mm; FOV 256 x 256 x 50 voxels) during the slower leakage phase of the contrast agent.^16,40^ Before contrast agent administration, 5 volumes of the slow and 3 volumes of the fast sequence were acquired. Next, during the fast sequence, gadolinium-based contrast agent (0.2 mL/kg = 0.1 mmol/kg gadoteric acid (Gd-DOTA, Clariscan) was intravenously injected in the antecubital vein (left side, if possible) with an injection rate of 3 mL/s and 25 mL saline flush). In advance, quantitative T1-mapping was performed to enable conversion of tissue signal intensity to gadolinium concentration.^40^ After DCE-MRI acquisition, the beforementioned FLAIR sequence was repeated (post-contrast FLAIR).

*Brain segmentation*

FLAIR and 3D T1-weighted images were processed using a whole-brain segmentation algorithm called sequence adaptive multimodal segmentation (SAMSEG) that is extendedwith the capability to handle white matter lesions,^41,42^ to segment the brain tissue volume (BV) into the following regions of interest (ROIs): gray matter (GM), normal-appearing white matter (NAWM) and white matter hyperintensities (WMH). WMH masks were visually checked and manually adjusted, if necessary (P.V.). Subsequently, brain-to-intracranial volume (BV/ICV) [-], as measure for atrophy, and WMH volume [cm^3^] were calculated from these masks.
